# Outbreaks of canine distemper in Dutch and Belgian mink farms

**DOI:** 10.1080/01652176.2018.1544427

**Published:** 2019-01-24

**Authors:** Robert J. Molenaar, Rianne Buter

**Affiliations:** GD Animal Health, Deventer, The Netherlands

**Keywords:** Mink, Neovison vison, canine distemper virus, molecular detection, wildlife

## Abstract

**Background:** Vaccination of farmed minks against canine distemper virus (CDV) has proved to be very effective. In the Netherlands, vaccination of farmed minks against CDV was mandatory until the closure of the local agricultural product boards at the end of 2014.

**Objectives:** To describe the first documented outbreaks of CD in Dutch mink farms since the closure of the agricultural product boards, as well as an outbreak in Belgium, with special attention to genotyping of the isolates.

**Methods:** A full post-mortem was performed on three carcasses per submission from farms A–C and on two carcasses from farm D. Molecular detection with subsequent typing was performed on eleven samples originating from four different farms. To assess genetic diversity partial sequences of the *H gene* of CDV were compared based on phylogenetic analysis.

**Results:** In 2017, there was a sudden series of CD outbreaks affecting four mink farms in the Netherlands (A–C) and Belgium (D). Gross, histologic and immunohistochemical findings were similar. There was a degree of genetic similarity between the viruses on farms A and D (98.5%) and between the viruses on farms B and C (97.3%), but the viruses from farms A and D belonged to a different clade than the viruses from farms B and C. Higher mortalities were reported in white and pastel minks.

**Conclusions:** Findings indicated that the difference in severity of the outbreaks was partially related to the genetic composition of the farm populations. Vaccination against CDV on Dutch and Belgian mink farms seems warranted.

## 1. Introduction

*Canine distemper virus* (CDV) is the highly contagious aetiology of the disease canine distemper (CD), which can cause high mortality on mink farms. Besides minks, the virus can infect and cause disease in a wide range of hosts including other members from the family *Mustelidae*, as well *Canidae*, *Procyonidae*, *Ursidae*, *Hyaenidae* and *Viverridae* and some members of the family *Felidae* (Deem et al. 2000).

Vaccination of farmed minks against CDV has proved to be very effective (Gorham 1999; Rikula et al. 2001). Indeed, CD is rarely diagnosed in commercially kept mink in those parts of the world where most farmers routinely vaccinate against the disease, such as North America (Wilson et al. 2015), even when the virus is considered enzootic in local wildlife (Roscoe 1993; Kapil and Yeary 2011). In the Netherlands, vaccination of farmed minks against CDV was mandatory until the closure of the local agricultural product boards at the end of 2014. During this time CD was an exceedingly rare disease in Dutch mink farms (unpublished data). CDV was detected in Northern European wildlife during this period (Renteria-Solis et al. 2014) posing a very real risk to farmed mink (Trebbien et al. 2014). In this article, we describe the first documented outbreaks of CD in Dutch mink farms since the closure of the agricultural product boards, as well as an outbreak in Belgium, with special attention to genotyping of the CD virus isolates.

## 2. Materials and methods

### 2.1. Case submission and follow-up

**Figure 1.**
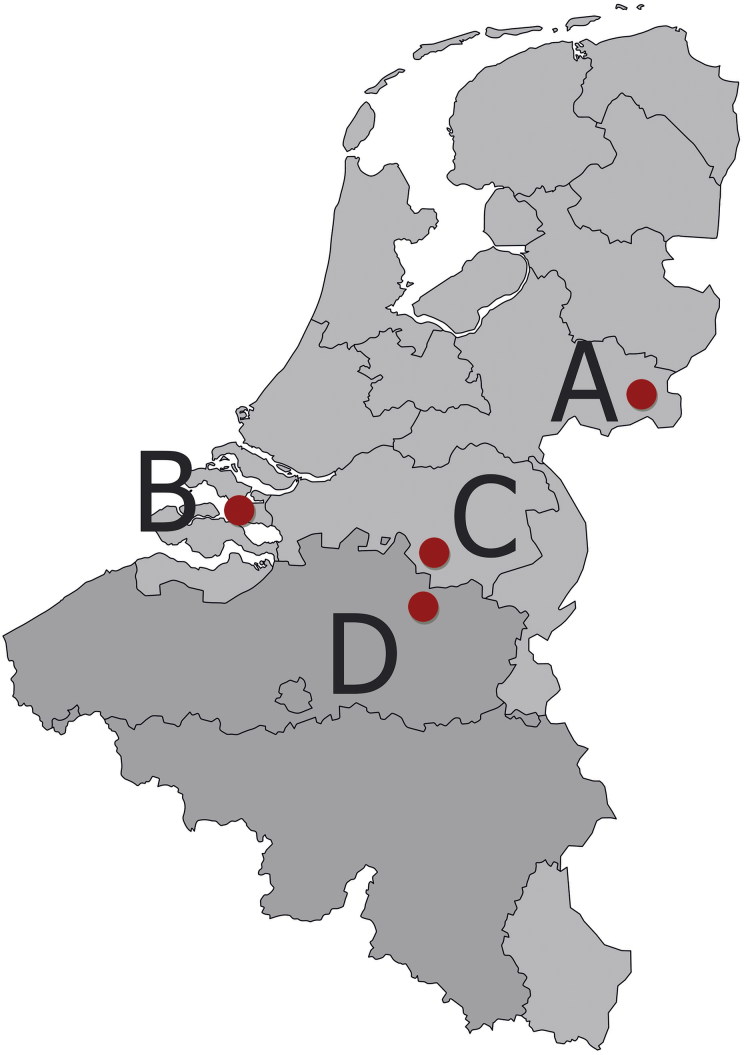
Map of the Benelux with the locations of farms A–D.

During 2017, carcasses from four commercial mink farms (A–D) suffering CD outbreaks were submitted to GD animal health services in Deventer, the Netherlands, for post-mortem analysis. Case A was submitted at the end of June, B at the end of September, C in the middle of October and D at the end of October. Farms A–C are in different parts of the Netherlands, and farm D is located in Belgium (Figure 1). In each case, the anamnesis mentioned thick footpads, crusts around the eyes or the mouth and a recent increase in mortality. A full post-mortem was performed on three carcasses per submission from farms A–C and on two carcasses from farm D, during which affected skin, lungs and either the urinary bladder or the kidneys were sampled in 4% buffered formalin for histological analysis and both the spleen and selected organs were stored at below –70 °C for molecular diagnostics and typing. From farm B the urinary tract was only sampled in two out of three minks, because of autolytic changes in the third one. Shortly after pelting time, all farms were interviewed by telephone, and again in April 2018, to assess the severity of losses and specifics of the clinical presentation.

### 2.2. Histological techniques

All formalin-fixed samples were paraffin-embedded within 48 h of collection, sectioned at 4 µm and stained with haematoxylin and eosin. From each sampled animal paraffin-embedded sections of skin, lung and urinary tract were additionally processed for immunohistochemistry (IHC) using CDV antibodies (Abnova, Taipei, Taiwan).

### 2.3. Molecular detection and genotyping

Molecular detection with subsequent typing was performed on 11 samples originating from four different farms. Two samples from farm A (lung and spleen), two from farm B (lung and spleen), two from farm C (lung and skin) and five from farm D (abdominal fat, gut contents, bile, liver and kidney). RNA was isolated from these samples with the MagMax Pathogen RNA/DNA isolation kit (4462359, Life Technologies, Carlsbad, CA) using the tissue protocol according to the manufacturer’s instructions.

Molecular detection was performed using the one-step real-time (RT) PCR kit (210212, Qiagen, Hilde, Germany) and the following protocol: reverse transcription at 50 °C for 30′ and 95 °C for 15′, followed by 40 cycles of denaturation at 95 °C for 30″, annealing and elongation at 56 °C for 30″ and 72 °C for 60″ and a final extension for 10′ at 72 °C. Purification and Sanger sequencing were performed using the amplicons of the PCR by Macrogen (Amsterdam, the Netherlands). For both PCR and sequence analysis a set of primers targeting the partial hemagglutination gene (*H gene*) was used as previously described (Zhang et al. 2017).

The sequences obtained in this study were aligned using MEGA 7 (Kumar et al. 2016). To gain insight into the genetic diversity of CDV partial sequences of the *H gene* of CDV were compared within and between farms. They were also compared with CDV *H gene* sequences reported previously (*n* = 35) (Zhang et al. 2017) and sequences deposited in Genbank (*n* = 103). Phylogenetic analysis was performed using BioNumerics version 7.6 (Applied Maths, Sint-Martens-Latem, Belgium).

**Figure 2.**
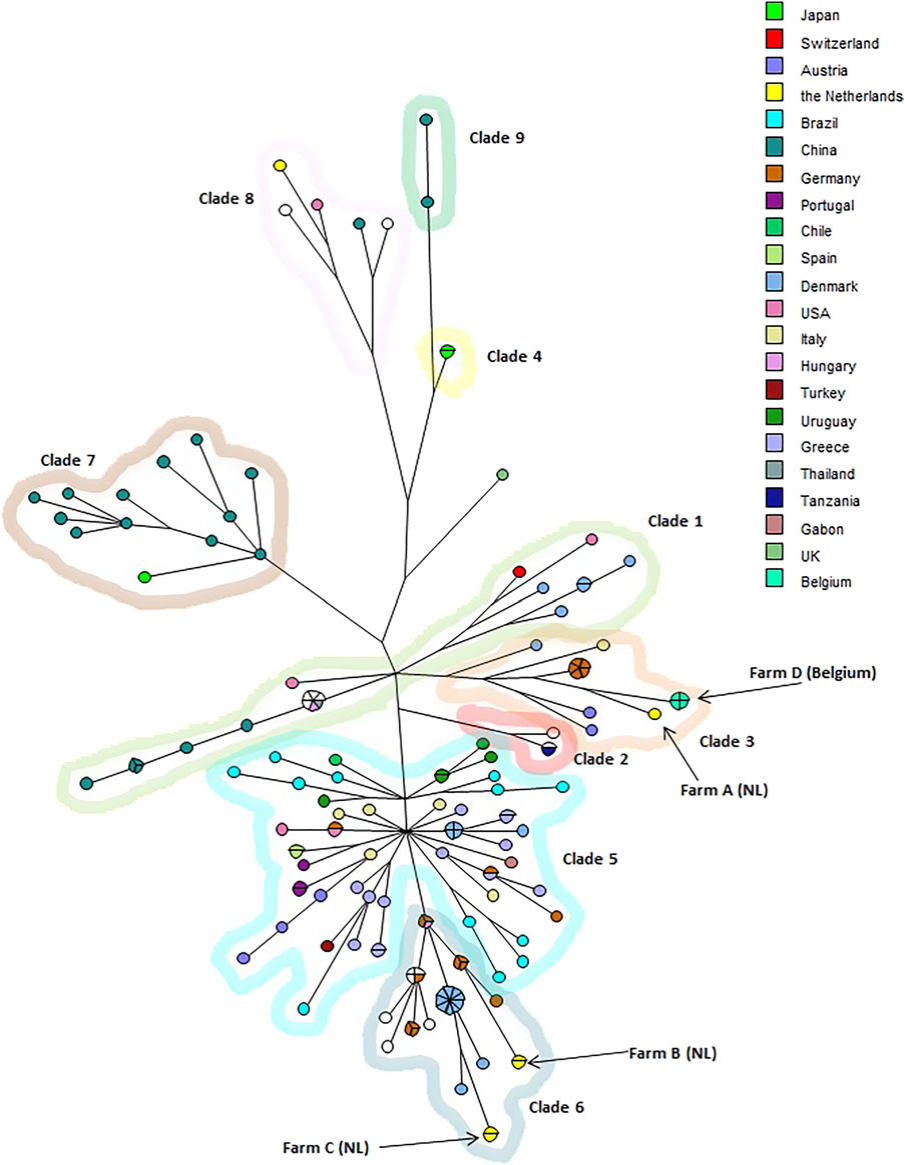
Phylogenetic analysis of the CDV *H gene* sequences from farms A–D, compared with a variety of sequences from CDV, colour-coded by country of origin. A single dot represents a single gene sequence, and bigger dots subdivided by lines represent multiple identical isolates.

From 148 CDV sequences originating from 21 countries and isolated from 17 different hosts a basic maximum parsimony tree was constructed (Figure 2). Clades were identified when two or more CDV isolates had a similarity of >96% at nucleotide level. These clades were compared with previously described genotypes (Zhang et al. 2017).

## 3. Results

### 3.1. Gross and clinical findings

All submitted minks showed macroscopic signs of hyperkeratosis and crusting on the face, mainly involving the regions around the eyes and the mouth. Classical signs of hyperkeratosis of the foot pads, which are the reason distemper is sometimes referred to as ‘hardpad disease’, as well as pneumonia were noted in farms B–D, but both were noticeably absent in animals submitted from farm A (Table 1). However, during a subsequent visit to farm A, a few minks with hyperkeratosis of the foot pads were noted.

**Table 1.** Number of examined animals with gross appearance of hyperkeratosis on footpads, facial area or elsewhere on the body and gross lesions of pneumonia as noted during post-mortem examination.

Farm	Hyperkeratosis foot pad	Hyperkeratosis around eyes or mouth	Hyperkeratosis elsewhere on body	Pneumonia
A	0/3	3/3	0/3	0/3
B	1/3	3/3	0/3	3/3
C	2/3	3/3	0/3	2/3
D	1/2	2/2	0/3	1/2

For each farm the population size and composition at the time of the outbreak, the country of origin of any animals imported in the last year, as well as some clinical specifics of the outbreak as noted by the farmers are presented in Table 2, based on interviews with the farmers. These interviews revealed that none of the kits on any of the farms were vaccinated against CDV and from the breeding animals only those at farm B were vaccinated against CDV. The owner of farm D concluded that, in hindsight, animals with typical CD lesions were already present at the end of June. This means that the chronological order of the outbreaks was A, D, B, C. Three of the farmers noted a difference in susceptibility between colour types. On farm B the white and silver-blue minks were clearly overrepresented in the mortality compared to the wild-type minks according to the farmer, but detailed data of losses was not available for analysis. On farm C the white minks were much more affected, suffering about 20% mortality, while farm average was only 2% (Table 2). Farm D mentioned that mortality was mostly limited to the pastel colour type, of which 30% died before the rest of the pastels was pelted down. Apart from these pastels, clear clinical signs of CD on farm D were only noted in a few albino minks. However, mortality in the other colour types on farm D was also mildly increased. There were no clinical signs of CD in these animals though and no further diagnostics were performed. Farms A, B and D started vaccinating their entire population against CDV as soon as they reached a diagnosis. Farm D also decided to pelt all their pastel mink, since roughly 30% of this colour type had already died since the start of the outbreak while mortality attributed to CD in the other colour types on the farm was incidental. Farm C did not vaccinate, since mortality was considered low and the disease occurred shortly before pelting time. The mink on farm C started to eat less, averaging at 120 g per day instead of 180 g. This was associated with a disappointing quality of the final product due to a considerable decrease in pelt size, decreased density of the underfur and a decreased percentage of pelts rated velvet, when compared to animals with the same genetic background that had remained at the farm from which the minks at farm C were originally imported. Both farm B and C had imported animals in the last winter, from Germany and Denmark respectively. When asked, the owners of the farms of origin said they didn’t experience a CD outbreak in 2017 or 2016.

**Table 2.** Population size and composition, country of origin of animals imported in the last year, as well as some clinical specifics of the outbreak as noted by the farmers.

Farm	Population size (number of breeding females)	Colour types present (% of population)	Import in last year (country of origin)	Mortality^a^	Treatment	Loss of pelt quality in survivors
A	4000	>98% brown <2% other colours	–	5%	Vaccination	–
B	8300	40% white 40% silverblue 20% brown	Germany	13%	Vaccination	Mild decrease in pelt size
C	2400	10% white 10% palomino 10% silver-cross 10% pearl 60% brown and silver	Denmark	2%	Pelting^b^	Mild to moderate decrease in pelt size Marked decrease in quality underfur Marked decrease in % velvet
D	1800	17% Pastel 83% Brown, silver-blue and albino	–	6%	Pelting of pastels Vaccination	–

a Mortality, including euthanasia, of minks with gross lesions suggestive of CDV as measured from start of outbreak until the pelting period.

b The outbreak occurred a few weeks before the normal pelting time, and the entire population was pelted before further escalation of the disease.

**Table 3.** Histological detection of inclusion bodies and CDV IHC positive staining in lung, skin and urinary tract samples.

Farm	Lung		Skin		Urinary bladder or kidney	
	Inclusion bodies	IHC positive	inclusion bodies	IHC positive	Inclusion bodies	IHC positive
A	3/3	3/3	0/3	3/3	3/3	3/3
B	3/3	3/3	2/3	3/3	0/2	2/2
C	3/3	3/3	0/3	3/3	0/3	1/3
D	2/2	2/2	0/2	2/2	2/2	2/2

### 3.2. Histological analysis

All examined lungs had an interstitial pneumonia with many intracytoplasmic eosinophilic inclusion bodies in bronchiolar epithelial cells (Table 3). In farm A these lung lesions were very mild. Skin samples had variable degrees of hyperkeratosis, occasionally with superficial bacterial infections. Two skin samples from farm B contained inclusion bodies in epidermis and follicular epithelium. Kidneys from farm A and urinary bladders from farms B, C and D were examined. The samples from farm A and D contained intracytoplasmic eosinophilic inclusion bodies in the transitional epithelium as well as mild degenerative changes.

IHC staining for CDV antigens was positive on all lung, skin and urinary tract samples, with the exception of two out of three urinary bladders from farm C.

### 3.3. Molecular detection and typing

Results of the phylogenetic analysis of the CDV *H gene* sequences are shown in Figure 2. Nine genetic clades could be defined, clade 5 being the largest clade with 56 CDV isolates, followed by clades 6 and 1 (32 and 22 isolates). Clades 7, 3, 8, 2, 4 and 9 consisted of 13, 11, 5, 3, 2 and 2 isolates, respectively.

Comparison of the CDV sequences of the four farms in this study showed that sequences within one farm were always identical. Between farms genetic variation was observed. Farms A and D showed some genetic relatedness (sequence similarity 98.5%) and clustered in clade 3 together with CDV isolates previously defined as the European wildlife genotype (Zhang et al. 2017). Also, some genetic relatedness between farms B and C was found (sequence similarity 97.3%). The sequences from these farms were found in clade 6 that seem to comprise of European strains of CDV. The sequence similarity between clade 3 (farms A/D) and clade 6 (farms B/C) was approximately 92%.

## 4. Discussion

Data that allow to assess the genetic diversity of CDV isolates in the Netherlands and Belgium are scarce. Although the CD viruses originating from farms A and D show high-sequence similarity, as do the CD viruses from farms B and C, hence they probably share a common ancestor. However, it cannot be concluded that the outbreaks are epidemiologically linked. Based on the sequence similarity the outbreaks on the four farms were the result of at least 2, but more likely four different CDV strains. Severity of the outbreaks appeared to be more related to the genetic composition of the mink population than to the genetics of the virus, with high mortality being reported in the white and especially pastel subpopulations. The sensitivity of pastel minks to CD has been previously documented (Appel 1987).

A possible explanation for the small sequence difference between CDV from farms A and D and from farms B and C would be infections from a shared reservoir, such as CDV sensitive wildlife which could spread the virus across the whole of the Netherlands (de Groot et al. 2016). The sequences from farms B and C are closely related to isolates from Germany (in raccoons in 2009) and Denmark (in minks in 2013), respectively. Interestingly, farm B had imported animals from Germany and farms C from Denmark, raising the question whether CDV was also introduced with these animals. The time between import of the minks and the CD outbreaks seemed unusual long though, especially considering the high number of unvaccinated kits in close contact with these imported animals. Additionally, there were no outbreaks on the farms from which the minks were imported even though kits on these farms were not vaccinated against CDV. The exact source of the outbreaks therefore remains unclear, and it is the authors hope that more extensive monitoring of Dutch wildlife and sequencing of possible CDV isolates might elucidate its role in CDV outbreaks in minks. In the meantime the implied role of wildlife, together with the recent detection of CDV in Belgian foxes (Garigliany et al. 2018), should be a reason to seriously consider vaccination against CDV on Dutch and Belgian mink farms but also in other sensitive species such as dogs. Indeed, Garigliany et al. already mentioned that the re-emergence of the virus is an argument to promote vaccination of domestic dogs against CDV. Spread of the virus from wildlife to domestic dogs has been discussed previously (Kapil and Yeary 2011). Transmission of CDV from wildlife to farmed minks by domestic dogs is an additional risk as they can have access to the farm area (Birch et al. 2017), suggesting that dogs of mink farmers should not just be vaccinated for their own sake.
